# Computational Analysis of the Interactions between the S100B Extracellular Chaperone and Its Amyloid β Peptide Client

**DOI:** 10.3390/ijms22073629

**Published:** 2021-03-31

**Authors:** Filipe E. P. Rodrigues, António J. Figueira, Cláudio M. Gomes, Miguel Machuqueiro

**Affiliations:** 1 Biosystems and Integrative Sciences Institute, Faculdade de Ciências, Universidade Lisboa, 1749-016 Lisbon, Portugal; ferodrigues@fc.ul.pt (F.E.P.R.); ajfigueira@fc.ul.pt (A.J.F.); 2 Departamento de Química e Bioquímica, Faculdade de Ciências, Universidade Lisboa, 1749-016 Lisbon, Portugal

**Keywords:** protein interactions, molecular dynamics, docking, chaperones, protein aggregation, amyloids, protein folding diseases

## Abstract

S100B is an astrocytic extracellular Ca^2+^-binding protein implicated in Alzheimer’s disease, whose role as a holdase-type chaperone delaying Aβ_42_ aggregation and toxicity was recently uncovered. Here, we employ computational biology approaches to dissect the structural details and dynamics of the interaction between S100B and Aβ_42_. Driven by previous structural data, we used the Aβ_25–35_ segment, which recapitulates key aspects of S100B activity, as a starting guide for the analysis. We used Haddock to establish a preferred binding mode, which was studied with the full length Aβ using long (1 μs) molecular dynamics (MD) simulations to investigate the structural dynamics and obtain representative interaction complexes. From the analysis, Aβ-Lys28 emerged as a key candidate for stabilizing interactions with the S100B binding cleft, in particular involving a triad composed of Met79, Thr82 and Glu86. Binding constant calculations concluded that coulombic interactions, presumably implicating the Lys28(Aβ)/Glu86(S100B) pair, are very relevant for the holdase-type chaperone activity. To confirm this experimentally, we examined the inhibitory effect of S100B over Aβ aggregation at high ionic strength. In agreement with the computational predictions, we observed that electrostatic perturbation of the Aβ-S100B interaction decreases anti-aggregation activity. Altogether, these findings unveil features relevant in the definition of selectivity of the S100B chaperone, with implications in Alzheimer’s disease.

## 1. Introduction

The regulation of protein aggregation by the protein quality control system is critical to main proteostasis in age-related protein deposition diseases such as Alzheimer’s disease (AD) [1]. The concerted action of chaperone repertoires in neurons counteracts the formation of misfolded aggregates, delaying the emergence of severe proteotoxicity. Ultimately, saturation of this machinery by accumulating misfolded proteins disables this protective system and neurodegeneration emerges. In AD, the extracellular accumulation of amyloid-β (Aβ) aggregates and the formation of senile plaques is one of the main prototypic disease hallmarks. It has been postulated that extracellular accumulation of misfolded Ab peptides starts several years before the appearance of disease symptoms.

During this prodromal stage, several responses to these early insults take place, including early neuroinflammation with the recruitment of glial cells and release of signaling mediators [2]. While most classical molecular chaperones operate inside cells, the regulation of extracellular proteostasis is limited to a few known examples that include clusterin, haptoglobulin and α2-macroglobulin, all of which are involved in multiple other functions [3]. Interestingly, that seems also to be the case for S100B, a member of the Ca^2+^-binding S100 family, which are multifunctional molecules [4,5]. In AD, S100B has so far been mainly associated with pro-inflammatory roles in late disease stages. However, recent evidence implicates this protein in multiple novel protective roles against Aβ aggregation and neurotoxic oligomer formation, including metal ion buffering scavenging [6,7] and the chaperone-like suppression of Aβ_42_ aggregation [8].

From a structural viewpoint, previous studies showed that Ca^2+^ binding to S100B prompts an interaction with Aβ_42_ monomers, oligomers and fibrils, which accounts for its inhibitory effect of both primary and surface-catalyzed secondary nucleation pathways of Aβ_42_ aggregation [8]. Structural analysis by two-dimensional NMR revealed that Aβ_42_ interacts with S100B at a binding cleft formed at the dimer interface, which opens upon Ca^2+^ binding to S100B. Surface hydrophobicity at this interface is amplified by Ca^2+^ binding to the four EF-hand domains, potentiating even more S100B interactions. In particular, this cleft involves two α-helices (helix IV), one from each of the S100B monomers, which comprise aggregation prone stretches [9,10] that display hydrophobic residues, further favoring homotypic interactions with aggregating peptides [11]. Analysis of the interaction by far-UV circular dichroism suggested that upon binding to Ca^2+^-S100B, monomeric Aβ_42_ partly adopts an α-helical conformation, a finding which could not be confirmed by NMR due to fast relaxation [8]. Therefore, many of the molecular details accounting for this regulatory interaction are still unknown.

Computational biology approaches have proven to be resourceful means to study biomolecular interactions when other methods of structural analysis provide incomplete information [12,13,14,15]. In the case of Aβ peptides, their lack of intrinsic structure in aqueous media, the fact that they gain different structures when interacting with specific partners, and their overall dynamic nature, makes these molecules very difficult to study using solution-based structural methods. Furthermore, several important intermolecular events involving Aβ, relevant to processes such as folding or binding, occur in a short time scale that is accessible with computational approaches. The complexity in these systems has also been holding back many computational efforts due to difficulties in obtaining adequate sampling [13,16]. Nevertheless, methods such as molecular dynamics (MD) simulations allow us to study the conformational space of many biomolecules at atomic resolution down to the µs timescale [13,15,17]. Also, these techniques can be complemented with molecular docking calculations, which provide starting configurations of macromolecular complexes that would otherwise take too long to obtain with MD simulations [18,19]. The increase in computational power combined with further developments in the used techniques will allow us to explore the dynamics of such systems over a wide range of time scales and promote further combinations of computational and experimental techniques [15].

In this work, we propose a concerted computational effort based on molecular docking and molecular dynamics simulations in order to identify and characterize the molecular details of the interaction between S100B and Aβ_42_. We showed that coulombic interactions are a major contributor to the maintenance and specificity of these complexes. Simulation outcomes suggesting the involvement of key electrostatic interactions were experimentally corroborated by testing the influence of high ionic strength in the inhibitory effect of S100B over Aβ_4_ aggregation. This work offers a detailed understanding of the interactions accounting for the chaperone activity of S100B, and paves the way to implement computational biology approaches to study the interactions with other partners.

## 2. Results and Discussion

### 2.1. Guided Docking of Aβ_42_ to S100B and Building of Interaction Complexes

**Figure 1:**
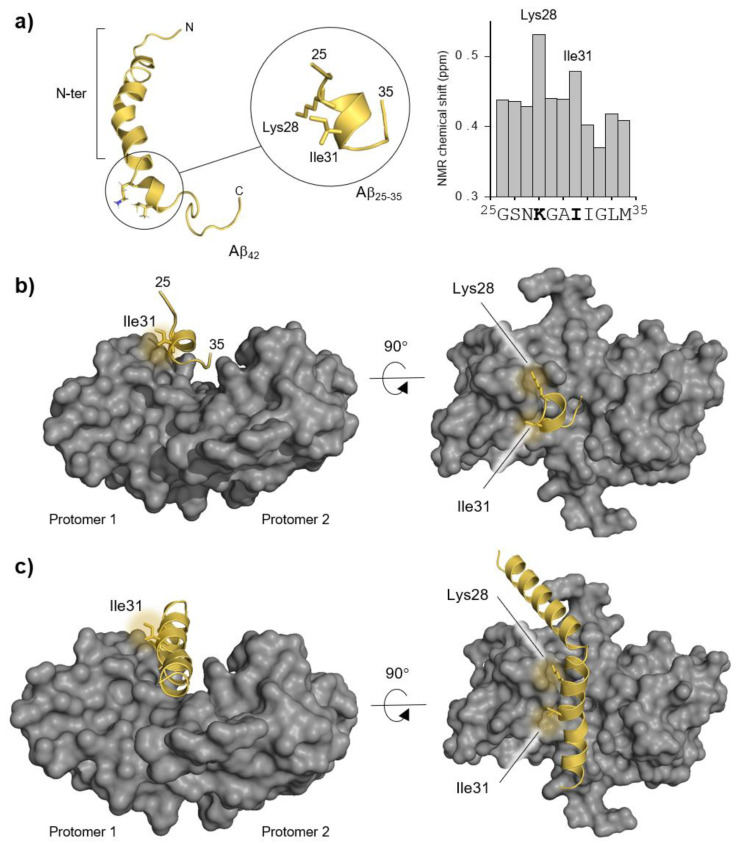
Molecular representations of Aβ42, Aβ_25–35_, S100B, and the main experimental data which guided the molecular docking procedure. (**a**) Aβ_42_ peptide cartoon structure (Protein Data Bank, PDB entry 1Z0Q) obtained from NMR in a 30/70 ratio solution of hexafluoro-2-propanol/water [22] (Lys28 and Ile31 residues are depicted in sticks to highlight their spatial proximity) and the NMR-based experimental evidence showing Lys28 and Ile31 interacting with S100B (redrawn from NMR interaction data from [8]). (**b**) Haddock solution used in this study with the Aβ_25–35_ structure located in the S100B (grey surface) binding cavity. (**c**) Full-length Aβ_42_ rebuilt from the Haddock solution in α-helical conformation. N-ter: N-terminus.

The Aβ_42_ peptide has a very complex conformational space that changes significantly with the solvent and the presence of interacting partners, thus resulting in enormous computational challenges [20]. Therefore, to determine the preferred binding modes to S100B, we employed the smaller Aβ fragment (Aβ_25–35_), which has simpler conformational and configurational spaces and is thus more amenable to analysis. This fragment was previously shown to interact with S100B, as the chaperone inhibits its aggregation in vitro and NMR evidenced significant chemical shifts in Aβ residues Lys28 and Ile31 upon titrating Aβ_42_ with S100B (Figure 1a) [21]. Altogether, this evidence shows that Aβ_25–35_ is an excellent starting model for the computational analysis as it recapitulates key aspects of S100B activity.

Nevertheless, this simpler starting model still allows us to recover interactions of Aβ_42_, which can be recovered by rebuilding from this shorter version. For this, we employed the Haddock online platform for molecular docking to study the configurational space between the Aβ_25–35_ peptide and the S100B protein. Following the experimental evidence indicating a partial conversion of Aβ to a helical conformation upon interaction with S100B [8], we performed the docking calculation starting from a peptide with the central core in a fully α-helical conformation. Haddock outputs were grouped in clusters and the ten best binding modes were selected by their interaction energies (Figure S1 and Table S1). Two binding modes were particularly promising since they were located at the binding cleft of S100B, which is the region known to interact with the Aβ peptides [8]. However, S100B is a homodimer, and these two binding modes are the mirror image of each other respective to the symmetry plane of S100B, which prompted us to proceed using only one of these structures (Figure 1b) to build the final complex. This binding mode also features both Lys28 and Ile31 residues facing the S100B interface, which is in excellent agreement with the experimental data. Interestingly, this cluster shows a very high electrostatic component to the Haddock scoring energy, hinting that electrostatic interactions may be pivotal in stabilizing the final complex. The complete Aβ_42_:S100B complex was created by rebuilding the remaining parts of Aβ_42_ in a helical configuration, pointing downwards towards S100B (Figure 1c).

Molecular dynamics simulations were then carried out to equilibrate the Aβ_42_ secondary structure, where the central interacting segment (25–35) was kept helical while the remaining peptide could freely sample the conformational space. The complex was also held together using distance and position restraints in these non-equilibrium equilibration steps. We created two specific segments of the Aβ peptide, labelled as the Aβ_25–35_ segment and N-terminus (N-ter) (Figure 1a) to simplify the discussion. The Aβ_25–35_ fragment comprises the more stable α-helix and contains Lys28 and Ile31, which are known to strongly interact with S100B, as previously mentioned. This is the portion of the peptide that we aimed to converge to equilibrium in the MD simulations. The N-ter region, which consists of Aβ residues 1–24, also interacts with S100B. However, since there is no evident guiding experimental data, we allowed the model to freely equilibrate while minimizing instabilities. It has been observed that this N-ter region has a lower propensity to form α-helical structures compared with the Aβ_25–35_ segment when lowering the dielectric of the solvent media [21]. Nevertheless, we opted to build this segment in full helical form at the start of the simulation to minimize our initial bias, since the rate of helix unfolding is significantly higher than that of helix formation. Indeed, at the end of the simulations after the equilibration procedure, most of the N-ter segment had lost its helical conformation, and the Aβ_42_ peptide exhibited conformations fully complementary to the S100B structure (Figure S2).

### 2.2. Molecular Dynamics Simulations and Mapping of Interactions

**Figure 2:**
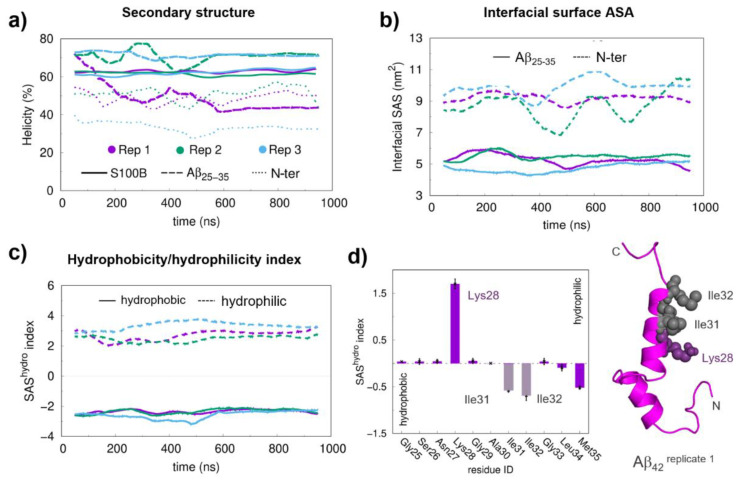
Structural equilibration properties, the hydrophobicity/hydrophilicity (SAS^hydro^) index, and the final conformations for the long molecular docking (MD) simulations. (**a**) Helical content of S100B, Aβ_25–35_ and Aβ_N-ter_ during production MD runs. (**b**) Interfacial area between Aβ_25–35_/Aβ_N-ter_ and S100B. (**c**) SAS^hydro^ indexes for the Aβ_25–35_ region at the interface. (**d**) Average SAS^hydro^ index values for the hydrophobic and hydrophilic residues in the Aβ_25–35_ segment. A floating window of 100 ns was used in the time series to reduce the local fluctuations. The final interfacial Solvent Acessible Surface Area (SASA) values were obtained by averaging the two interfacial areas, one mapped on the protein and another mapped on the peptide surface. The hydrophobic and hydrophilic residues in the SAS^hydro^ index calculations were separated based on their sign (positive for hydrophilic and negative for hydrophobic residues). The average SAS^hydro^ index values were obtained from the equilibrated segments (the last 450 ns). The error bars were calculated from the standard error of the mean between replicates. A representative structure of the Aβ_42_ peptide is shown in the cartoon with the most relevant residues, in terms of SAS^hydro^ index values, represented with spheres.

We next investigated the stability of the Aβ_42_:S100B complexes from long (1 μs) MD simulations to determine trajectories and preferred binding modes. To assure convergence of several structural properties during the simulations, we calculated the variation of the helical content of the Aβ_25–35_ and N-ter regions, as well as of the S100B protein (Figure 2a). The secondary structure of both S100B and Aβ_25–35_ was found to be very stable throughout the simulations, with Aβ_25–35_ being nearly fully helical, especially in replicates 2 and 3 (Figure S3). The secondary structure of the N-ter region of Aβ_42_ also converged for all replicates, suggesting that the system is near equilibrium.

We then determined the interface contact area between S100B and Aβ_42_ to characterize the molecular details of the interaction (Figure 2b). The results obtained showed that the interfacial area of the Aβ_25–35_ region tended to stabilize at around 5 nm^2^ for all replicates, without significant deviations throughout the simulations, an indication that the formed interactions are stable. Thus, these results rule out major configurational changes or the possibility that the Aβ_25–35_ segment detaches from S100B during the simulations, indicating that the binding modes between Aβ_42_ and S100B are relatively homogeneous. The larger fluctuations in the interface area of the N-ter region indicate that the interface established by this segment has increased dynamics, even though it converges after ~500 ns of simulation.

Next, we mapped the residues at the Aβ_25–35_:S100B interface and monitored the evolution of their involvement in interfacial contacts during the simulation (Table S2 and Figure S4). Interfacial residues within the Aβ_25–35_ segment vary discretely along the simulation while S100B undergoes an interfacial change during the first 550 ns, prior to the formation of the final interaction surface. Therefore, the initial 550 ns of all replicates in the simulations were discarded whenever equilibrium properties were calculated to assure equilibration of the properties associated with the Aβ_25–35_ region. We then determined the hydrophobicity/hydrophilicity index (SAS^hydro^) [7], which corresponds to the computed solvent-accessible surface area [23] of each residue weighted by its hydrophobicity Wimley–White score [23]. This allowed us to decompose the relative contribution of hydrophobic (negative SAS^hydro^) and hydrophilic (positive SAS^hydro^) interactions established in the Aβ_42_:S100B complex. Analysis of the Aβ_25–35_ segment shows a balanced contribution between hydrophobic and hydrophilic residues along the simulation (Figure 2c), indicating an amphiphilic character. Inspection of the SAS^hydro^ index for the individual residues in the Aβ_25–35_ segment shows matching positive and negative contributions from hydrophilic (Lys28) and from hydrophobic (Ile31, Ile32, and Met35) residues that cancel each other out (Figure 2d). The prominent contribution of Lys28 suggests that its role in the formation of the Aβ:S100B complex likely involves electrostatic interactions.

**Figure 3:**
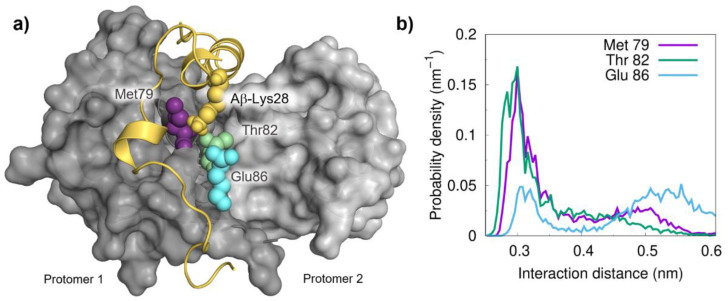
Mapping the interactions of Lys28 from Aβ42 with S100B residues. (**a**) Structural representation of the Aβ_42_:S100B complex highlighting the important role of Lys28 (yellow) and distances to nearby residues Met79 (pink, 0.74 nm), Thr82 (green, 0.37 nm) and Glu (blue, 0.58 nm). (**b**) Probability density distributions of minimum distances between Lys28 (the terminal amino group) and the hot spot partner residues on the S100B surface.

Following the detection of a significant role for Aβ-Lys28 in the interaction, we next developed a procedure to identify preferential contacts based on a distance threshold (0.35 nm) between this residue and those in S100B. Using this approach, the analysis of the final equilibrated complexes allowed us to identify three residues that persistently interact with Lys28: Met79, Thr82 and Glu86. All these residues are located on helix IV at the S100B binding cleft, and this ensemble likely forms a diffuse binding hotspot (Figure 3a). In agreement, the calculated probability density distributions obtained from the simulations perfectly capture these interactions (Figure 3b). Interestingly, the presence of Glu86 at the interaction interface suggests that this residue in S100B likely acts as an electrostatic anchor to stabilize the interaction with Aβ via its Lys28.

### 2.3. Energetics of the Interaction between Aβ and S100B and Implications in Chaperone Activity

**Figure 4:**
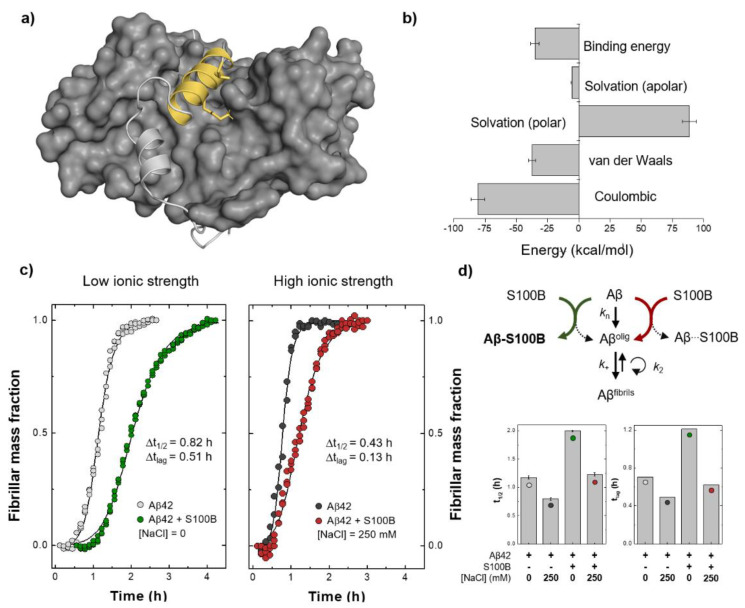
Estimation of the binding energy for the Aβ:S100B complex and experimental evidence supporting the relevance of coulombic interactions for Aβ:S100B complex stabilization. (**a**) Structural representation of the Aβ:S100B complex (depicted as light grey cartoon with Aβ_25–35_ in yellow, on the protein grey surface) highlighting the contributions of Lys28 and Ile31 residues (marked with sticks). (**b**) Molecular mechanics Poisson–Boltzmann surface area (MM-PBSA) binding energy calculated for the Aβ_25–35_:S100B complex as the sum of all energetic terms involved over the equilibrated segments of the simulations and with the error bars calculated from the standard error of the mean between replicates. (**c**) Aggregation of 5 µM Aβ_42_ at 37 °C with or without 25 µM Ca^2+^-S100B under low (left) and high (right) ionic strength conditions. (**d**) Binding impairment between S100B and monomeric Aβ_42_ at high ionic strength accounts for the partial depletion of S100B inhibitory activity, for example, over the mechanism of primary nucleation, which is exclusively dependent on monomeric Aβ_42_ concentration (top). Half-time (t_1/2_) and lag time (t_lag_) values of Aβ_42_ aggregation in all tested conditions (error bars represent standard deviation, n = 3) (bottom).

We carried out a quantitative analysis of the different contributions to the binding energy of the Aβ:S100B complex. We employed a methodology based on molecular mechanics Poisson–Boltzmann surface area (MM-PBSA) analysis, focusing on the Aβ_25–35_ segment. In this analysis, despite some variability, the energy terms reasonably converged after 550 ns of simulations (Figure S5) and the calculation of the different energetic terms evidence electrostatics as the main driver for binding. The results show that the interaction between the Aβ_25–35_ segment and S100B (Figure 4a) is indeed energetically favorable (−35.0 kcal/mol) and that there is a significant contribution from coulombic stabilization (−80.8 ± 5.5 kcal/mol) (Figure 4b).

Finally, we set out to experimentally explore the potential role of coulombic interactions on S100B:Aβ_42_ complex stabilization. We tested the inhibitory effect of S100B over Aβ_42_ aggregation under low and high ionic strength conditions (Figure 4c). Indeed, at a five-fold excess ratio, we observed that S100B anti-aggregation activity, measured by an increase in the time required for the formation of 50% of fibrillar mass (t_1/2_), was significantly lower in the presence of 250 mM NaCl when compared with the appropriate controls in the absence of salt (Figure 4d). Since NaCl itself displayed an accelerating effect on Aβ_42_ aggregation, the S100B activity decrease might be better depicted by the difference (Δt_1/2_) in the increasing t_1/2_ due to the presence of the inhibitor (Figure 4c inset). The ionic strength-dependent depletion of S100B activity was even more remarkable when we compared the lag/pre-transition times (t_lag_)—here defined as the highest measured time where the fibrillar mass was lower than 10%—in both conditions (Figure 4c inset and Figure 4d). Lag phase duration is highly dependent on the rates of primary and secondary nucleation, as well as fibril elongation [24]. Moreover, a decrease in the concentration of free monomeric Aβ_42_ as caused, for instance, by binding to an inhibitor, may strongly affect the rates associated with all these mechanisms. This dependence is especially noticeable in the primary nucleation reaction, which comprises the association and structural conversion of monomers in solution to form the first smallest fibrillar aggregates that can be further extended by elongation [25]. A minor t_lag_ change associated with a lower transition/exponential phase slope in the presence of an aggregation inhibitor, as observed for S100B under high ionic strength conditions, is typical of the quasi-selective inhibition of secondary nucleation promoted by the interaction of the chaperone with fibril surfaces, and not monomers [26]. Therefore, our results also suggest that S100B association with Aβ_42_ fibrils is driven by a different set of intermolecular forces that can be established even at high ionic strength. Altogether, and accounting for the fact that S100B holdase activity requires, at least partly, proper interaction with its client peptide in the monomeric state, the aggregation kinetic outcomes support the meaningful role of coulombic interactions for the formation and maintenance of S100B:Aβ_42_ complexes as suggested by molecular simulation experiments (Figure 4d, top).

## 3. Materials and Methods

### 3.1. Materials and Protein Purification

All reagents were of the highest grade commercially available. Thioflavin T (ThT) was obtained from Sigma and sodium chloride (NaCl) was obtained from NZYTech (Lisboa, Portugal). A chelex resin (Bio-Rad) was used to remove contaminant trace metals from all buffers. Recombinant Aβ_42_ was expressed in *E. coli* and purified as described [27]. To obtain the monomeric form, 2.5 mg of Aβ_42_ was dissolved in 7 M guanidine hydrochloride (Sigma) and eluted in a Superdex S75 (GE Healthcare) with 50 mM HEPES (4-(2-hydroxyethyl)-1-piperazineethanesulfonic acid) pH 7.4. Monomeric Aβ_42_ concentration was estimated by UV spectroscopy (Shimadzu UV-1700) at 280 nm using the theoretical extinction coefficient value of ε_280 nm_ = 1424 M^−1^cm^−1^. Low-binding tubes (Axygen Scientific, Corning) were used in all manipulations of Aβ_42_. Human S100B was expressed in *E. coli* and purified to homogeneity as described [28]. Apo-S100B was prepared by incubation at 37 °C for 2 h with a 300-fold excess of dithiothreitol (DTT) and 0.5 mM EDTA (ethylenediamine tetraacetic acid) and eluted in a Superdex S75 (GE Healthcare, Oeiras, Portugal). S100B concentrations are expressed as homo-dimer equivalents.

### 3.2. Aβ_42_ Aggregation Kinetics

Thioflavin T (ThT) aggregation kinetics were evaluated by recording the ThT fluorescence intensity in a plate reader (Fluostar Optima, BMG Labtech, Ortenberg, Germany) with a 440 nm excitation filter and a 480 nm emission filter. The fluorescence was recorded using bottom optics in half area 96-well polyethylene glycol-coated black polystyrene plates with a clear bottom (Corning, 3881). Monomeric Aβ_42_ was diluted in 50 mM HEPES pH 7.4 supplemented with 1.1 mM CaCl_2_ to a final concentration of 5 µM with the indicated concentrations of S100B and NaCl. ThT (10 μM) was added to each condition. The assays were performed at 37 °C, under quiescent conditions and fluorescence measurements taken every 400 s. Aggregation kinetics data were normalized and fitted using the AmyloFit online platform [29] by the secondary nucleation dominated model [30].

### 3.3. Molecular Docking Settings

The S100B dimer obtained from the crystal structure available in the Protein Data Bank (PDB: 3D0Y [31]) was stripped of water and the last two missing residues from each monomer were added using PyMOL (Version 2.0, Schrödinger, LLC, San Diego, CA, USA). The Aβ peptide from residues 25 to 35 (Aβ_25–35_) was built in a helical conformation using PyMOL. In water, this segment is in equilibrium between the folded (helix) and unfolded state. Following the experimental evidence, we postulate that the helical conformations should be the most stable and abundant when interacting with S100B. The docking calculations were done using the Haddock web server 2.2 with standard parameters. All 11 residues of the Aβ segment were assigned as active residues, while a selected list of residues from S100B, obtained from experimental data (Table S3) [8], was assigned as active, with passive residues assigned automatically. Two of the best Haddock solutions were in agreement with the experimental evidence regarding the S100B interaction region (Figure S1). Additionally, since S100B is a homodimer, the two solutions are mirror images of each other, which leads to only one binding mode to be studied using molecular dynamics simulations.

### 3.4. Molecular Mechanics/Molecular Dynamics Settings

All simulations were performed with GROMACS v.2018.6 [32,33] and the GROMOS 54a7 force field [34,35]. We identified three distinct metal coordination centers (Figure S6) in the S100B dimer (PDB: 3D0Y [31]), which were parameterized at the quantum level. These centers were constituted by all residues coordinated with metals, through their side or main chains. All missing hydrogen atoms were added, and all residues coordinated by their main chains were mutated to Ala residues to simplify our Quantum Mechanics (QM) model. The final structures were geometry optimized using Gaussian [36] with B3LYP functional and 6-31G basis set [37], from which the missing bonded parameters were obtained. The van der Waals radii used for calcium and zinc ions were 0.24 and 0.21 nm [38], respectively. The partial charges were obtained from electrostatic potential calculations using the Merz–Kollman scheme and manually curated to adhere to the GROMOS charge group convention [39]. It is worth noting that the numbering of the S100B residues follows a specific order; the residues from the second monomer are numbered as n + 94, to account for both the 91 residues from the first monomer its respective 3 metal coordination sites, which can be seen on the topology file (available as Supporting Material). The full Aβ peptide (Aβ_42_) was rebuilt from the Aβ_25–35_ segment in the Haddock solution pose. The remaining residues were built in a helical conformation with a kink resembling the experimental NMR structure [22]. By setting this helical conformation in the initial structure, we adopted the smallest bias available since the process of partially unfolding an α-helix peptide is faster, and within our computational reach, than partially folding a fully disordered structure. The corrected protein dimer (see the previous section) interacting with the Aβ peptide were solvated with 15,000 Single Point Charge (SPC) water molecules in a dodecahedral box with a minimum distance of 1.2 nm between the protein and the limits of the box. The protonation states of all residues were chosen according to their respective abundance at physiological pH levels (only Asp, Glu, Lys, and Arg were ionized). To achieve charge neutrality in the system, 19 Na^+^ ions were added. A v-rescale heat bath at 310 K with separate couplings for the solute (protein content) and solvent (water and ions) and a relaxation time of 0.1 ps was used to handle temperature coupling. Parrinello–Rahman isotropic pressure coupling was used to keep the pressure of the system at 1 bar, with isothermal compressibility of 4.5 × 10^−5^ bar^−1^, and a relaxation time of 0.5 ps. The LINCS algorithm was used to constrain all protein bonds. The SETTLE algorithm was used to constrain all water molecules. The equations of motions were integrated with a 2 fs time step, with the list of neighbors being updated every 10 ps. The Particle Mesh Ewald electrostatics treatment method was used [40] with 0.12 nm for the maximum grid spacing of the Fast Fourier Transform, an interpolation order of 4, and a cutoff distance of 1.4 nm for Lennard–Jones and Coulomb interactions. The systems were energy minimized in two steps (~30,000 steps each) using the steepest descent algorithm, one unconstrained and following one constrained with the pLINCS algorithm applied to all bonds [41]. The initialization procedures took place in three steps: first, a 100 ps NVT MD simulation with initial velocities generated from a Maxwell velocity distribution at 310 K and position restraints of 1000 kJ mol^−1^ nm^−2^ on all C_α_ atoms was performed. This was followed by a 200 ps NPT simulation with the Parrinello–Rahman isotropic pressure couple and weaker position restraints of 100 kJ mol^−1^ nm^−2^. Lastly, a 200 ps NPT simulation like the previous one, with weaker position restraints, of 10 kJ mol^−1^ nm^−2^ was carried out. A 200 ns pre-equilibration MD simulation with distance restraints on the Aβ_25–35_ helical segment was performed to optimize the interface between the peptide and the protein. Three replicates of 1 µs each were produced, starting from three different positions of the pre-equilibration run. Equilibrium properties and statistical analyses were performed on conformations obtained after the equilibration step, which we determined to occur after 550 ns. Nevertheless, to better evaluate the convergence of the simulations, all time-series included the complete data (1 µs).

### 3.5. Computational Analyses

All analyses were performed using GROMACS tools and/or in-house scripts. A series of analyses based on solvent accessible surface area (SASA) [23] were employed. The interfacial area of the S100B–Aβ complexes was obtained by calculating the SASA of the S100B protein in a complex both in the presence and absence of the Aβ partner. A difference between these 2 values in each conformation provided the desired interfacial area. This analysis was also done for each residue separately, leading to the hydrophobicity/hydrophilicity index [7]. This was calculated by summing the product between the interfacial SAS and the hydrophobicity (Wimley–White) scale [23] of each residue. The information can then be averaged out over time and plotted per residue or can be averaged out over residue and plotted over time. In this work, a minimum distance protocol was implemented in order to identify interacting residues from S100B with Lys28 of Aβ_42_. The minimum distance between each residue of S100B and the nitrogen atom of the side chain of Lys28 of Aβ_42_ was calculated. An initial cutoff of 0.6 nm was used to discard weak or non-existent interactions and histograms with the joined data from the three replicates were generated for the more relevant residues. It was also possible to evaluate strong interactions, which corresponded to a percentage of time from the equilibrated segment of the simulations, where each residue was within a 0.35 nm distance. The free energies of binding for the S100B:Aβ_25–35_ complex were calculated using the molecular mechanics with Poisson–Boltzmann and surface area (MM/PBSA) solvation method [42]. Here, the binding energy was estimated from the sum of four distinct energetic terms: E_coul_ was calculated with a Coulomb potential and E_VdW_, which is calculated with a Lennard–Jones potential, both in a vacuum; to take into consideration solvation effects, two additional terms are estimated: Solv_Polar_ to account for electrostatic interactions with the solvent, and Solv_Apolar_ to take into consideration the energetic cost of creating a cavity in the solvent (as well as the apolar interactions with solvent). All calculations were performed by our own code (https://github.com/mms-fcul/mmpbsa; accessed on 1 February 2021), using G54A7 compatible charges and radii, a dielectric constant of 4 [43], and an ionic strength of 0.1 M. All error values in properties averaged over the equilibrated segments of the 3 replicates were estimated using the standard error of the mean. Images were rendered using PyMOL.

## 4. Conclusions

Understanding the interactions established between the S100B chaperone and its amyloid β client is critical to characterize the mechanistic aspects underlying its holdase activity and to evaluate effects on amyloid aggregation pathways. Here, we resorted to computational and experimental approaches to provide compelling evidence that the S100B protein displays structural features enabling a dynamic association with Aβ in its monomeric form. The simulations support previous experimental findings, showing that Aβ can be stabilized upon interaction with S100B along a stable interaction interface formed at a crevice between the two protomers that constitute the functional dimer, and support the evidence for partial folding upon binding with α-helix formation within the Aβ_25–35_ segment. These chaperone–client interactions comprise a strong coulombic component between glutamates in helix IV from S100B and Aβ_42_ Lys28, which provide selectivity for an otherwise hydrophobic-driven interaction, a finding corroborated by a decreased inhibitory efficiency at high ionic strength. Altogether, this work reports important mechanistic advances of S100B function and illustrates the added value of combining experimental and computational approaches.

## Data Availability

All data are presented in the manuscript and are available upon reasonable request.

## Supplementary Materials

The following are available online at https://www.mdpi.com/article/10.3390/ijms22073629/s1. Figure S1: Structural representation of the ten best Haddock solutions for the Aβ_25–35_:S100B complex. Figure S2: Molecular representations of the Aβ_42_ bound to S100B in the final configurations after the equilibration MD step. Figure S3: Molecular representations of the Aβ_42_ bound to S100B in the final configurations after production MD. Figure S4: The interface percentage for S100B and Aβ_25–35_ calculated using the initial or the final conformations as a reference. Figure S5: The different energetic contributions to the MM-PBSA binding energy of the Aβ_25–35_:S100B complex over time. Figure S6: Structural representation of the three distinct metal coordination sites on S100B. Table S1: Haddock scores and energy contributions from the Haddock calculations. Table S2: Summary table with the key residues involved in the interface of the Aβ_25–35_:S100B complex. Table S3: S100B residues defined as active for the Haddock calculations. Supplementary Files: Force field, topology, and conformation files.
