# Supplementary material for: Computational Analysis of the Interactions between the S100B Extracellular Chaperone and Its Amyloid β Peptide Client
Source: Int J Mol Sci. 2021 Mar 31;22(7):3629. doi: 10.3390/ijms22073629 (PMC8037576; doi:10.3390/ijms22073629)
Supplement: Supplementary file 1 [file ijms-22-03629-s001.pdf]

Supplementary Materials

Computational analysis of the interactions between the S100B extracellular chaperone and its amyloid β peptide client

Filipe E. P. Rodrigues[1,2], António J. Figueira[1,2], Cláudio M. Gomes[1,2*], Miguel Machuqueiro[1,2*]

1 Biosystems and Integrative Sciences Institute, Faculdade de Ciências, Universidade Lisboa, Lisbon, Portugal; E-Mails: ferodrigues@fc.ul.pt (F.E.P.R), ajfigueira@fc.ul.pt (A.J.F), cmgomes@fc.ul.pt (C.M.G.), machuque@fc.ul.pt (M.M.)

2 Departamento de Química e Bioquímica, Faculdade de Ciências, Universidade Lisboa, Lisbon, Portugal

List SI materials:

Figure S1. Structural representation of the ten best Haddock solutions for the $A\beta_{25\text{-}35}$:S100B complex.
Figure S2. Molecular representations of the $A\beta_{42}$ bound to S100B in the final configurations after the equilibration MD step.
Figure S3. Molecular representations of the $A\beta_{42}$ bound to S100B in the final configurations after production MD.
Figure S4. The interface percentage for S100B and $A\beta_{25\text{-}35}$ calculated using the initial or the final conformations as a reference.
Figure S5. The different energetic contributions to the MM-PBSA binding energy of the $A\beta_{25\text{-}35}$:S100B complex over time.
Figure S6. Structural representation of the three distinct metal coordination sites on S100B.

Table S1. Haddock scores and energy contributions from the Haddock calculations.
Table S2. Summary table with the key residues involved in the interface of the $A\beta_{25\text{-}35}$:S100B complex.
Table S3. S100B residues defined as active for the Haddock calculations.

Supplementary Files: Force field, topology, and conformation files (SuplementaryFiles_Rodrigues.zip).

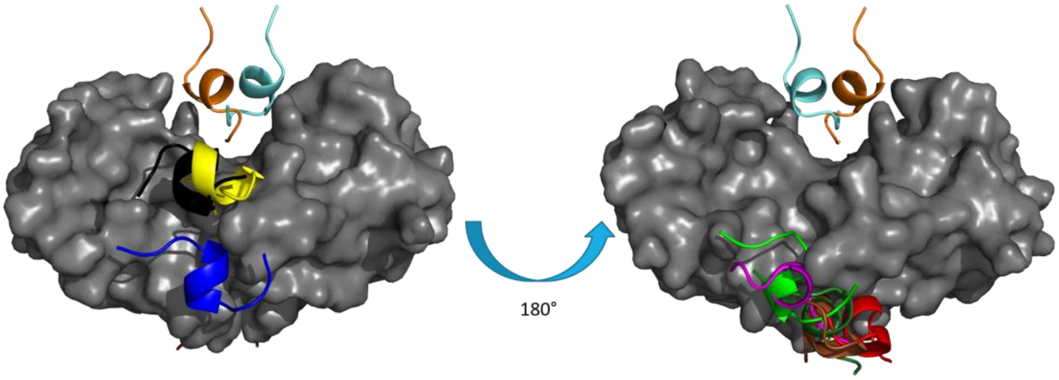

Figure S1. Structural representation of the ten best Haddock solutions for the Aβ25-35:S100B complex. In the right panel, the image is rotated 180 degrees. The Aβ25–35 peptide structures are shown in cartoons colored in different colors, while the S100B is depicted in a grey surface. The colors magenta (1), green (2), red (3), black (4), blue (5), light blue (6), brown (7), orange (8), dark green (9), and yellow (10) follow the numeric order of the clusters in Table S1. Both light blue and orange solutions (clusters 6 and 8) present the peptide in the binding cavity, in perfect agreement with the experimental evidence [7]. In fact, these two solutions only represent one binding mode since, due to the symmetry axis of S100B homodimer, they are the mirror image from each other.

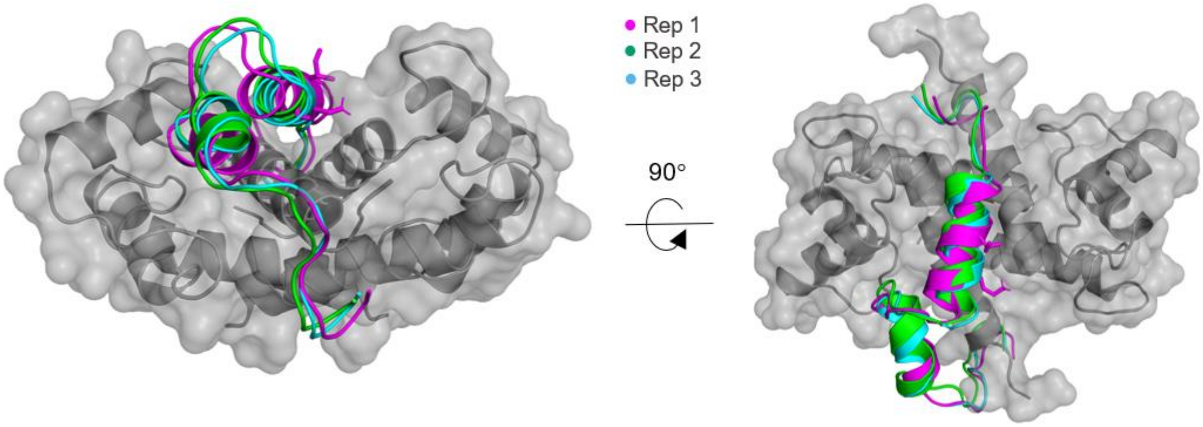

Figure S2. Molecular representations of the Aβ42 bound to S100B in the final configurations after the <u>equilibration MD step</u>. The 3 structures, corresponding to different times (180, 190, and 200 ns), were used as the starting configurations of each replicate (each Aβ42 color represents a different replicate). The S100B is shown in grey cartoon and transparent grey surface.

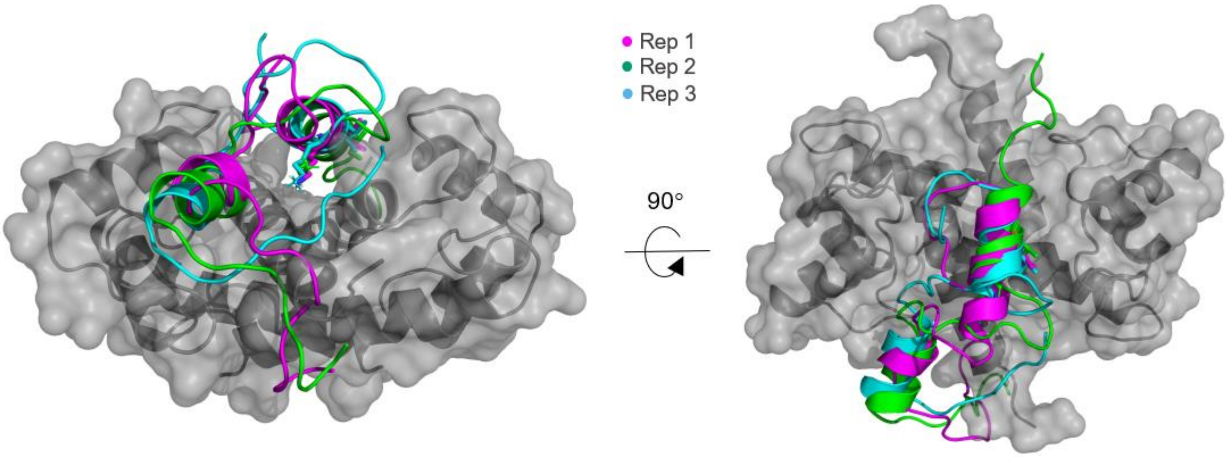

Figure S3. Molecular representations of the Aβ42 bound to S100B in the final configurations after <u>production MD</u>. The 3 $A\beta_{42}$ structures in cartoon representation with different colors correspond to the 3 replicates used. The S100B is shown in grey cartoon and transparent grey surface.

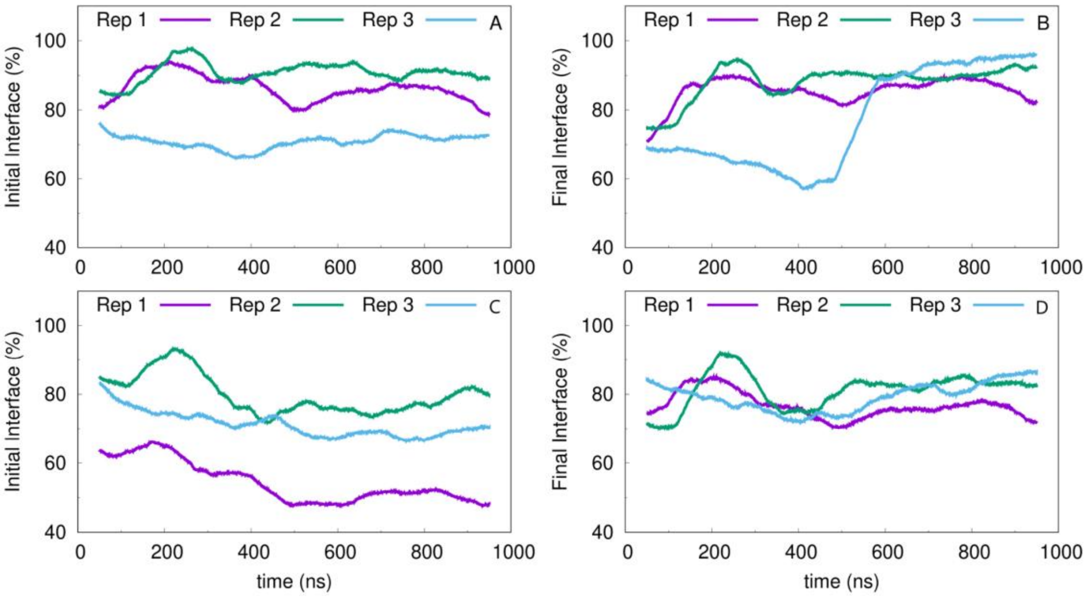

Figure S4. The interface percentage for S100B (A and B) and $A\beta_{25-35}$ (C and D) calculated using the initial (A and C) or the final conformations (B and D) as a reference. The percentages were calculated using a sum of the interface area of each identified residue involved in the interface in the start or end of the simulations (see Table S2). A floating window of 100 ns was used in order to reduce the local fluctuations of each data series. Panel B shows how the peptide shifts location in the S100B cavity, a process that was fast for the first two replicates, but it took over 500 ns in the third replicate.

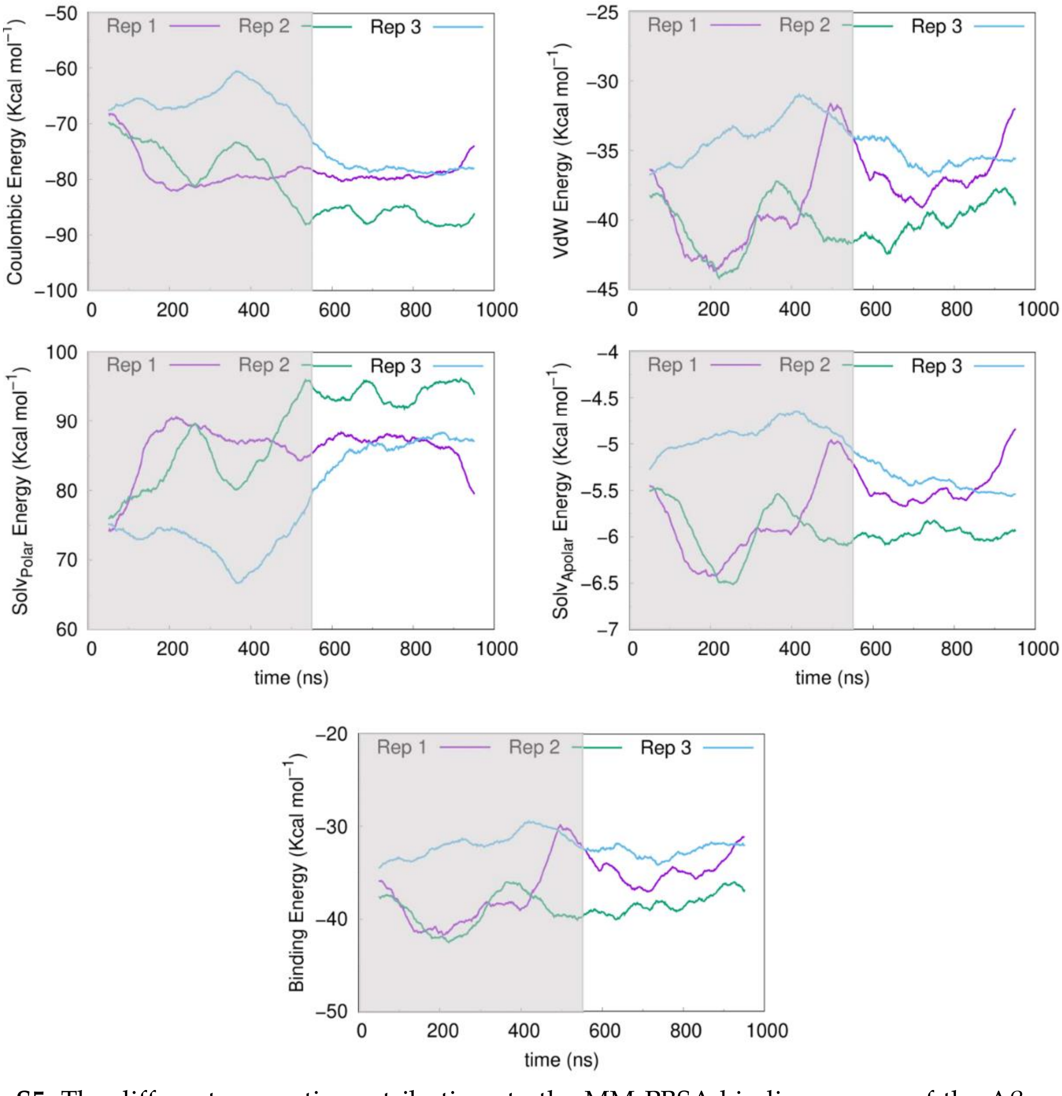

Figure S5. The different energetic contributions to the MM-PBSA binding energy of the $A\beta_{25-35}$:S100B complex over time. The Coulombic and Van der Waals (VdW) energies are the vacuum molecular

mechanics contributions. The Apolar ($Solv_{Apolar}$) and the Polar ($Solv_{Polar}$) refer to the solvation energy contributions. The sum of these 4 terms leads to the binding free energy of the complex. The greyed out region corresponds to the non-equilibrated portion of our simulations. The balance between the Coulombic contribution and the $Solv_{Polar}$ penalty are the two main components which drive the binding energy of the complex.

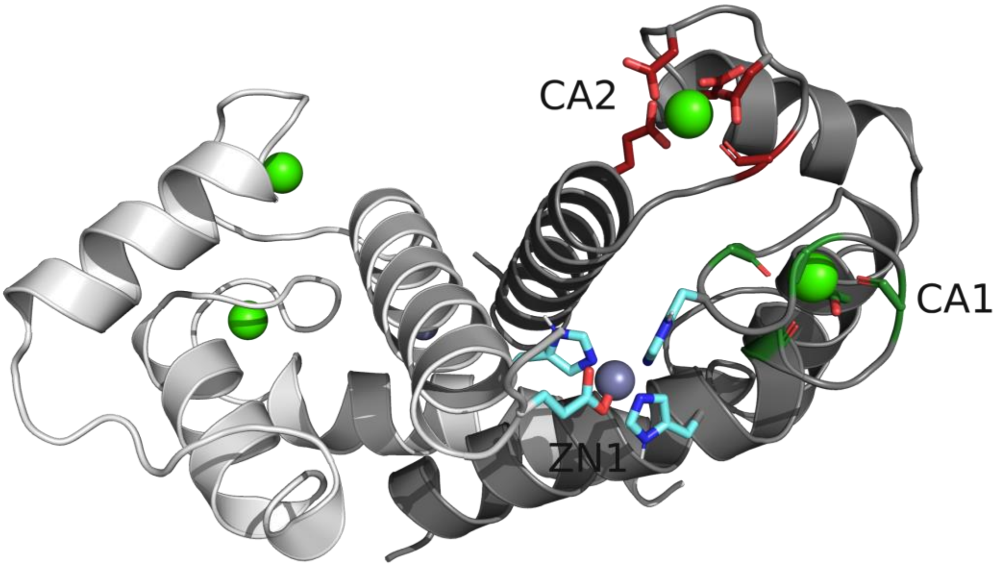

Figure S6. Structural representation of the three distinct metal coordination sites on S100B. The protein is depicted in cartoon, with the monomers colored with light and dark grey. In some metal coordination sites, the residues are marked with sticks and calcium and zinc ions are represented with green and dark grey spheres, respectively. There are two calcium centers with distinct geometries (red or green sticks) and one zinc (cyan sticks). A second set of these binding sites exists on the other S100B monomer. The zinc binding sites share ligands between both monomers.

Table S1. Haddock scores and energy contributions from the Haddock calculations. The clusters were ordered by their Haddock Score. The Haddock run generated only two seemingly symmetrical solutions (clusters 6 and 8) located on the S100B binding cavity. Due to this symmetry, only cluster 6 was used in the system building.

Cluster	$E_{elec,}$ (Kcal/mol)	E_{VdW} (Kcal/mol)	Haddock Score
1	-59	-31	-70
2	-64	-28	-64
3	-90	-24	-59
4	-54	-31	-57
5	-51	-27	-55
**6**	**-134**	**-21**	**-52**
7	-91	-22	-50
**8**	**-138**	**-16**	**-43**
9	-45	-19	-43
10	-32	-19	-43

Table S2. Summary table with the key residues involved in the interface of the Aβ_{25-35}:S100B complex. These were identified using SASA interface calculations for each residue in the initial and final conformations of each replicate. Residues marked with a * are part of a metal binding site in S100B.

Species	Key Residues	
	Initial interfacial residues	Final interfacial residues
S100B	Asn62, Gln71, Met74, Ala75, Ala78, Met79, Thr82, Thr153, Gly160, Cys162, Glu166*, Phe170	Gln71, Ala75, Met79, Thr82, Thr153, Leu154, Cys162, Glu166*, Ala169, Phe170, Met173
Aβ_{25-35}	Gly25, Lys28, Gly29, Ile31, Ile32, Gly33, Leu34, Met35	Gly25, Ser26, Lys28, Ile31, Ile32, Met35

Table S3. S100B residues defined as active for the Haddock calculations. This selection was based on the experimental evidence pinpointing the S100B region that interacts with Aβ_{42} [7]. Residues marked with a * are part of a metal binding site in S100B.

S100B active residues	Asn62, Gln71, Met74, Ala75, Ala78, Met79, Thr82, Ala83, Gln165, Glu166*, Ala169, Ala172, Met173, Thr176, Glu180

Supplementary Files: Force field, topology, and conformation files (SuplementaryFiles_Rodrigues.zip).

All files can be found as extra SI or in this direct link:

http://mms.rd.ciencias.ulisboa.pt/files/FilipeRodrgues_G54A7-ModFF.zip
